# Tumor hypoxia modulates podoplanin/CCL21 interactions in CCR7+ NK cell recruitment and CCR7+ tumor cell mobilization

**DOI:** 10.18632/oncotarget.16311

**Published:** 2017-03-17

**Authors:** Anna Tejchman, Nathalie Lamerant-Fayel, Jean-Claude Jacquinet, Aleksandra Bielawska-Pohl, Katarzyna Mleczko-Sanecka, Catherine Grillon, Salem Chouaib, Maciej Ugorski, Claudine Kieda

**Affiliations:** ^1^ Centre for Molecular Biophysics, UPR 4301 CNRS affiliated to Orléans University and INSERM, Orléans, France; ^2^ Laboratory of Glycobiology and Intercellular Interactions, Institute of Immunology and Experimental Therapy, PAN, Wroclaw, Poland; ^3^ ICOA, UMR CNRS 7311, University of Orleans, Orleans, France; ^4^ INSERM U1186, Gustave Roussy Institute, Villejuif, France; ^5^ Malopolska Centre of Biotechnology, Jagiellonian University, Krakow, Poland

**Keywords:** adhesion, cancer associated fibroblasts, CCL21, hypoxia, podoplanin

## Abstract

Podoplanin (PDPN), an O-glycosylated, transmembrane, mucin-type glycoprotein, is expressed by cancer associated fibroblasts (CAFs). In malignant transformation, PDPN is subjected to changes and its role is yet to be established. Here we show that it is involved in modulating the activity of the CCL21/CCR7 chemokine/receptor axis in a hypoxia-dependent manner. In the present model, breast cancer MDA-MB-231 cells and NKL3 cells express the surface CCR7 receptor for CCL21 chemokine which is a potent chemoattractant able to bind to PDPN. The impact of the CCL21/CCR7 axis in the molecular mechanism of the adhesion of NKL3 cells and of MDA-MB-231 breast cancer cells was reduced in a hypoxic tumor environment. In addition to its known effect on migration, CCL21/CCR7 interaction was shown to allow NK cell adhesion to endothelial cells (ECs) and its reduction by hypoxia. A PDPN expressing model of CAFs made it possible to demonstrate the same CCL21/CCR7 axis involvement in the tumor cells to CAFs recognition mechanism through PDPN binding of CCL21. PDPN was induced by hypoxia and its overexpression undergoes a reduction of adhesion, making it an anti-adhesion molecule in the absence of CCL21, in the tumor. CCL21/CCR7 modulated NK cells/ECs and MDA-MB-231 cells/CAF PDPN-dependent interactions were further shown to be linked to hypoxia-dependent microRNAs as miRs: miR-210 and specifically miR-21, miR-29b which influence PDPN expression.

## INTRODUCTION

In the tumor microenvironment and in the secondary lymphoid organs, CCL21 chemokine is a potent and specific chemoattractant upon presentation on the surface of endothelial cells via glycosaminoglycans (GAGs) [1]. In non-transformed cells, it is constitutively expressed by high endothelial cells in lymph nodes and Peyer's patches, lymphatic ECs as well as stromal cells in the spleen and appendix [2]. Its receptor, CCR7, is present on subpopulations of T cells [3], semi-mature and mature dendritic cells (DCs) [4], natural killer (NK) and natural killer T (NKT) [5, 3] cells. CCL21 interaction with its transmembrane receptor activates integrins which mediate the firm adhesion of leukocytes to endothelium [6]. CCL21, together with CCL19 and the highly O-glycosylated, mucin-type glycoprotein called podoplanin (PDPN) are required for normal lymphoid tissue organization. This is essential for effective T cell-DC interactions [7, 8, 9]. Lymphatic ECs secrete CCL21 in a complex, together with PDPN which was shown to attract CCR7+ cells [10]. Tumor growth inhibition was evidenced upon CCL21 expression in colon carcinoma and murine models of melanoma as well as mammary carcinoma [12, 15, 16]. CCL21-mediated help of tumor regression was shown to depend on host T and NK cell activity [11].

CCR7 is also expressed by non-immune cells, most notably in malignancies [12]. In many tumor types, including MDA-MB-231 breast cancer cells, expression of CCR7 drives metastases into lymph nodes [13]. This also occurs in melanoma [14, 15], colorectal cancer [16, 17], non-small cell lung cancer [18], and chronic lymphocytic leukemia [19].

In these malignancies, interaction of CCR7 with CCL21 drives migration and invasion of CCR7^+^ cancer cells into lymph nodes similarly to normal homing [18, 20]. Furthermore the effect of CCR7 on tumor progression in primary mouse and human breast tumors is mediated through stem-like cells by decreasing their ability to self-renew and triggering neoplasia [21].

Podoplanin expression has been studied in a variety of cancer cells in relation to lymphatic vasculature [22]. *In vivo*, its overexpression in MCF-7 breast cancer cells promotes metastasis into lymph nodes [23]. It induces platelet aggregation with circulating tumor cells, which helps metastasis [24, 25]. In mammary tumors, cancer associated fibroblasts (CAFs) which constitute a major cell component of the stroma, express PDPN but little is known about its mechanism of expression [26, 27] and modulation. This may be crucial for the movement of cancer cells, more specifically cancer stem cells which differently express cell adhesion molecules [28], for their ability to escape the tumor site. According to this theory a key role in the control of tumor microenvironment is played by microRNAs (miRs) on gene expression occurring post-transcriptionally [29]. Among others, miR-21 is a regulator of the oncogenic process since it regulates cell proliferation, survival and migration of most cancer cells through its target proteins [30], mainly the tumor suppressor PTEN (phosphatase and tensin homologue deleted on chromosome ten) the activity of which correlates with miR-301 [31]. Since PTEN controls tumor growth and metastasis by regulating tumor angiogenesis [32] it controls the hypoxic status inside the tumor, thereby possibly alleviating hypoxia in tumors.

Therefore, the aim of the present study was to analyze the molecular mechanisms of the recognition of CCR7+NK cells and microvascular ECs in order to decipher their role in the NK cells recruitment into tumors. Moreover, the effect of CCL21 availability to breast cancer cells, through PDPN expression by CAFs, was addressed. PDPN is shown to be modulated by hypoxia and related to miR-21, miR-210 and miR-29b expression in CAF-like fibroblasts. This work demonstrates that tumor hypoxia, by modulating the expression of molecules such as PDPN, leads to easier liberation of tumor cells and, lowering the NK cells recruitment upon CCL21 presentation, it impairs the anti-tumor immune defence. These studies, conducted under conditions mimicking the tumor microenvironment show the fundamental influence of hypoxia on PDPN expression by fibroblasts and its presentation of CCL21 to CCR7+ tumor cells and CCR7+ NK cells.

## RESULTS

### CCR7 expression and cell adhesion properties of NK cells to endothelial cells from peripheral lymph nodes

**Figure 1:**
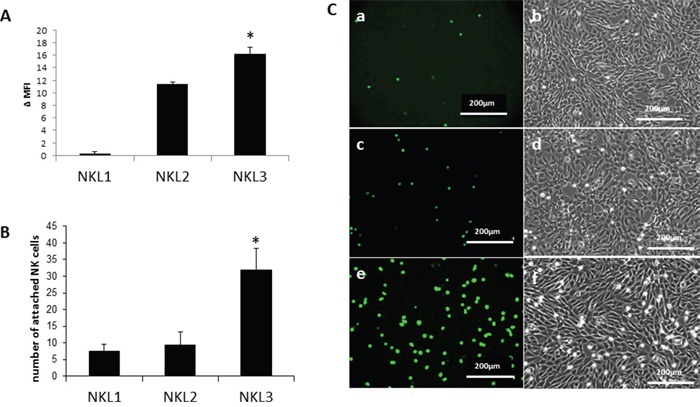
Adhesion of NK cell lines on endothelial cells from peripheral lymph nodes (HPLNEC.B3 cells) **A**. Expression of the CCL21 receptor (CCR7) on NKL1, NKL2 and NKL3 cells surface. Results represent Δ MFI (mean fluorescence intensity) from flow cytometry analysis. Values marked with a star vary significantly (p<0.05, N=5) from the NKL1 level of expression. **B**. Quantification of adhered NK cells counted from ten representative fields. Values marked with a star vary significantly (p<0.05, N=3) from NKL1 and NKL2 adhesion levels. **C**. Flow adhesion data of NKL cell lines on HPLNEC.B3 cells NKL1 (a, b); NKL2 (c, d); (e, f).

Three different NK cell lines [33, 34] were characterized for CCR7 expression on their surface (Figure 1). NKL1 cells did not express CCR7. In contrast, NKL2 and NKL3 cells expressed the receptor; NKL3 displayed a higher level of expression than NKL2 cells (Figure 1A). Figure 1B provides the quantification of the adhesion of the NK cell lines, tested on human peripheral lymph node endothelial HPLNEC.B3 cells [35] under flow conditions, which correlates with CCR7 expression on the NK lines (Figure 1A, 1B). Figure 1C shows that NKL3 adhered more efficiently to HPLNEC.B3 cells under flow (Figure 1C e, f) when compared to NKL1 (Figure 1C a, b) and NKL2 (Figure 1C c, d). However, the latter cell line adheres more efficiently than NKL1 cells.

### The influence of CCL21 activity and hypoxia on adhesion properties of NKL3

**Figure 2:**
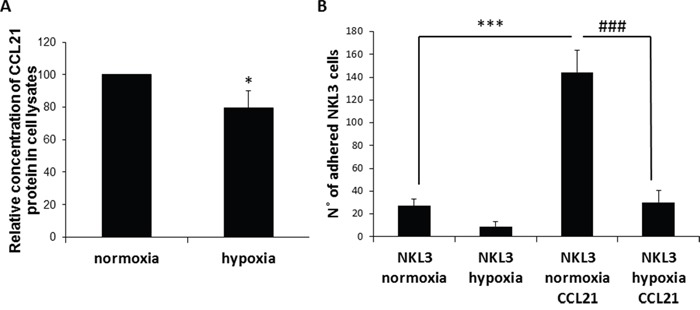
Influence of CCL21 presentation by peripheral lymph node endothelial cells and hypoxia on NK cells recognition and adhesion **A**. Hypoxia vs normoxia CCL21 production by HPLNEC.B3 cells measured by ELISA in cell lysates. Concentration of CCL21 protein produced under normoxia was set as 100%. Values marked with a star vary significantly (p<0.05, N=3). **B**. Quantification of the NKL3 adhesion to HPLNEC.B3 as number of NKL3 cells counted on the surface of HPLNEC.B3 cells (ten representative fields were counted) in normoxia and in hypoxia showing a reduction before as well as after treatment with CCL21. NK cells were labelled by the PKH26 red fluorescent cell linker kit. NK cells were injected on the HPLNEC.B3 cells monolayer (1.10^6^ NK cells/ml) at a fixed flow rate of 0, 2 dyn/cm^2^ for 5 minutes and washing by OptiMEM at 0, 6 dyn/cm^2^ for 10 min. Adhered NK cells amount was quantified. *** p<0.001, N=3, ### p<0.001, N=3.

CCL21 is known as a chemoattractant for many immune cells, in particular NK cells. The mechanism of attraction is potentially endothelial cell-mediated. To assess the possible changes in chemokine production by endothelial cells under hypoxia, CCL21 was quantified by ELISA in the cell lysates of HPLNEC.B3 cells cultured under normoxia or hypoxia (Figure 2A). It appeared that significantly less CCL21 was produced under hypoxia than normoxia although its mRNA expression was highly increased under hypoxic conditions (Supplementary Figure 1). This may possibly influence the recruitment and adhesion level of NK cells on hypoxic endothelial cells.

The role of ECs CCL21 was assessed by pre-incubation of HPLNEC.B3 cells in the presence of exogenous CCL21 prior to the measurement of NKL3 cell adhesion. Under normoxic control conditions, the adhesion level of NKL3 cells to endothelial cells when pre-stimulated by CCL21 was 6.5 times higher than towards non stimulated cells (Figure 2B). Hypoxia is one of the main characteristics of tumor microenvironments. Its influence on NK cell adhesion properties was investigated. Hypoxia is known to regulate some adhesion molecule expression, and this can especially be observed on HPLNEC.B3 cells. This could explain the observed adhesion decrease [34, 36]. But, another hypothesis could be put forward based on the expression and activity of CCL21 which is the main chemokine involved in cell recruitment into lymphoid organs.

To mimic intra tumor conditions HPLNEC.B3 cells were submitted to hypoxia (1%) for 24 hours prior to the adhesion assay testing for the involvement of CCL21. Figure 2B shows that NKL3 cells had 70% less adhesion to hypoxia-treated HPLNEC.B3 than in normoxia. Upon treatment by CCL21 the adhesion level was increased 6 fold although it remained 4.5 times lower than with CCL21 treatment in normoxia. This shows the effect of tumor microenvironment conditions on the efficacy of the antitumor immune cells.

### Capping of CCR7 receptor induces an increase of NKL3 cell adhesion

**Figure 3:**
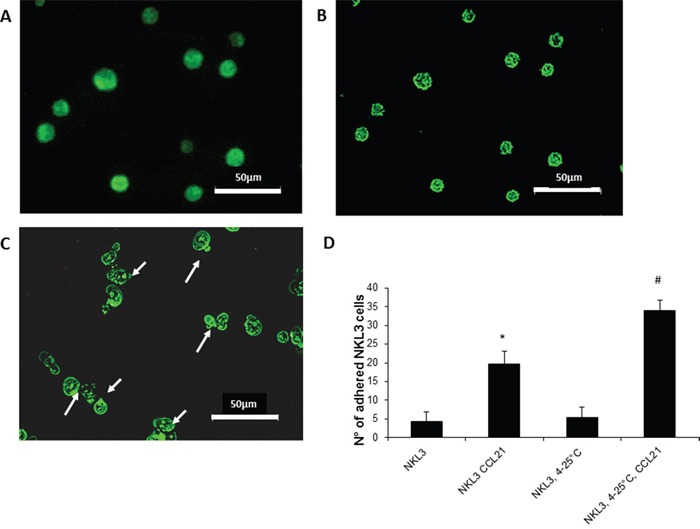
CCR7 detection, dynamics and activity on NKL3 cells **A**. NK cells labelled with FTC-anti-CCR7 antibodies, at 4°C and fixed with 4% PFA, capping does not occur. **B**. Patching upon warming the cells from 4°C to 25°C for 5 min. at 25°C. **C**. Warming for 15 mins, capping occurs. **D**. Quantification of the NKL3 cells adhesion to HPLNEC.B3 as number of NKL3 cells counted on the surface of HPLNEC.B3 cells monolayer (ten representative fields were counted), NKL3 were either maintained at room temperature or capping was induced (4-25°C) before and after preincubation with CCL21 *p<0.05, **p<0,01, N=3, # p<0.05, ## p<0,01 N=3.

The adhesion process is highly dependent on cell energy and metabolism. To distinguish the pathways by which CCR7 might be involved in adhesion/recognition, its dynamic status was checked on NKL3 cell membranes. When cells were exposed to mouse anti-CCR7 FITC coupled antibodies at 4°C and then fixed with PFA, the receptor was found dispersed on the plasma membrane (Figure 3A). However, when cells were similarly labelled at 4°C and then at room temperature, i.e. 25°C, for 5 minutes, CCR7 concentrated into patches (Figure 3B). After 15 min. incubation at 25°C, receptors clustered into polar caps (Figure 3C, arrows).

The dynamic redistribution of CCR7 on the cell surface greatly influenced the adhesion properties of NKL3 cells. When NKL3 cells were first treated by CCL21 a considerable effect on the adhesion efficacy was observed (Figure 3D) and when CCL21 receptor-CCR7 capping induction was permitted by a temperature jump from 4°C to 25°C (15 min.), NKL3 cells adhered more efficiently on HPLNEC.B3 cells (Figure 3D). This indicates the role of CCL21 and the dynamics of its receptor in the adhesion process of NK cells to endothelial cells.

### CCR7-CCL21 axis role in the recognition mechanism of NKL cells to HPLNEC.B3 cells

**Figure 4:**
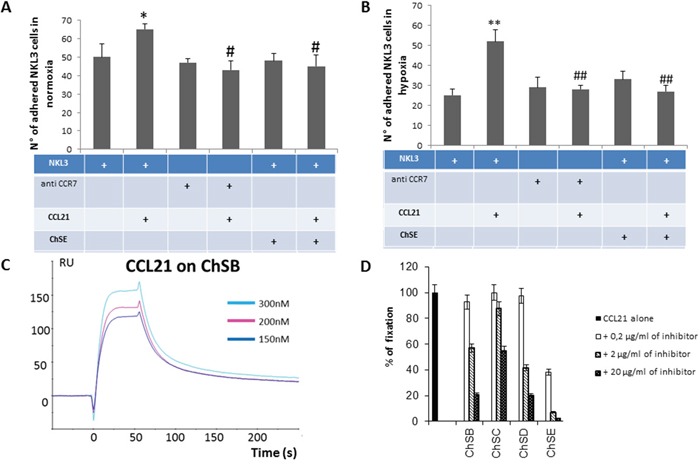
**A**. Impact of CCL21 presentation by GAGs on CCR7+ NK cells adhesion to endothelial cells. Quantification of the NKL3 adhesion to HPLNEC.B3 as number of NKL3 cells counted on the surface of HPLNEC.B3 cells (ten representative fields were counted) in normoxia (A) and in hypoxia **B**. showing an increase in adhered NKL3 after treatment with CCL21 and decrease after treatment with CCR7 neutralizing antibody and chondroitin sulfate in both conditions. NK cells were labelled by PKH26 red fluorescent cell linker kit then, treated or not with CCR7 neutralizing antibodies (10 μg/ml), or ChSE (0,6 μg/ml) for 1 hour at 4°C. Then, NK cells were injected on the HPLNEC.B3 cells monolayer (1.10^6^ NK cells/ml in OptiMEM) at a fixed flow rate of 0, 2 dyn/cm^2^ for 5 minutes and washing by OptiMEM at 0, 6 dyn/cm^2^ for 10 min. Adhered NK cells amount was quantified. * p<0.05, N=3, # p<0.05, N=3; ** p<0.005, N=3, ## p<0.005, N=3. **C**. Plasmon resonance assessment of the affinity of CCL21 chemokine for various glycosaminoglycans. CCL21 was injected over the GAG surface in a range of concentrations (from 0 to 300 nM) to produce sensorgrams for association and dissociation phases analysis. Binding kinetics were evaluated with a 1:1 Langmuir model using the BiaEvaluation software (Biacore). **D**. Inhibition experiments by co-injection of CCL21 chemokine with various concentrations of GAGs on the immobilized ChSD surface. Relative inhibition was preferential for chondroitin sulphate E compared to ChS B, D and C.

As described above, when HPLNEC.B3 cells were pre-incubated with exogenous CCL21, the adhesion level of NK cells was strongly increased confirming previous studies [34, 6]. To decipher the molecular mechanism of the interaction, HPLNEC.B3 cells were pre-incubated with exogenous CCL21 showing a 30% increase of the NKL3 numbers of adhered cells (Figure 4A) in normoxia. This adhesion increase by CCL21 was prevented when NKL3 cells were pre-incubated with anti-CCR7 neutralizing antibodies. Then the adhesion of NKL3 cells remained at the basal control level (obtained when NKL3 were incubated with anti-CCR7 antibodies). This confirmed the CCR7/CCL21 implication in the increase of NK cell adhesion to endothelial cells. Moreover, if HPLNEC. B3 pre-incubation with CCL21 was performed in the presence of chondroïtin sulfate E, the adhesion increase was abrogated while incubation of HPLNEC.B3 cells with only chondroitin sulfate E did not affect adhesion compared to control.

These experiments were performed under hypoxic conditions so the endothelial cells could mimic the tumor microenvironment (Figure 4B). It was confirmed that NKL3 basic adhesion was reduced when compared to normoxia. CCL21 treatment of HPLNEC.B3 induced a high increase of the adhesion (2.5 fold). Similar to data obtained under normoxic conditions, CCR7 involvement in NKL3 cells was shown by the total inhibition obtained upon blocking the receptor with anti CCR7 neutralizing antibodies. Moreover chondroitin sulfate E-mediated inhibition of increase of adhesion due to CCL21, was total, indicating chemokine presentation by endothelial glycosaminoglycan. In hypoxia, this is accompanied by an increase of the chondroitin sulfate expression by HPLNEC.B3 cells (up to 71% by 48 H) as shown by A. Selo (doctoral thesis).

To characterize the chemokine CCL21 interaction with glycosaminoglycans of the chondroitin sulfate family, a direct binding assay was performed by surface plasmon resonance. The CCL21 binding to fixed chondroitin and concentration dependency is shown in Figure 4C, while the specificity for the E form of chondroitin sulfate is demonstrated in Figure 4D. This shows a competitive inhibition of the binding of CCL21 to chondroitin sulfate B (high molecular weight) by synthetic tetrasaccharides of the ChE type compared to tetrasaccharides of the D, B and C forms of chondroitin sulfate.

### CCL21/CCR7 implication in tumor cell adhesion to fibroblasts expressing podoplanin

**Figure 5:**
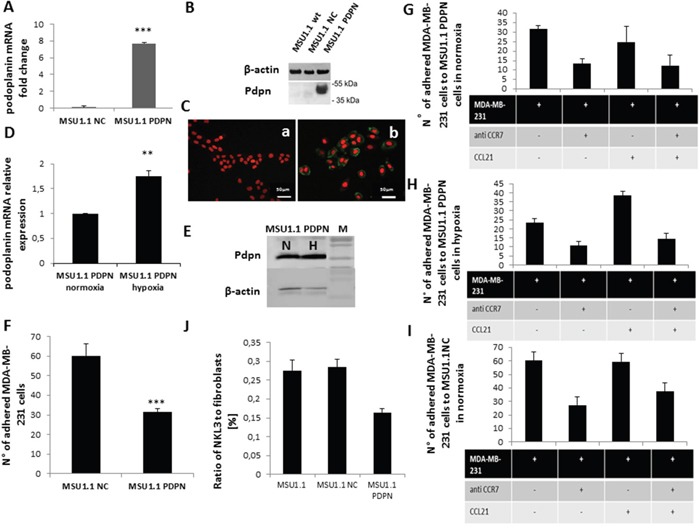
Impact of podoplanin/CCL21 interaction on MDA-MB-231 cells adhesion to MSU1.1 and MSU1.1 PDPN cells surface in normoxia and hypoxia **A**. Quantification of podoplanin expression at mRNA level in hypoxia vs normoxia. **B**. PDPN protein detection by immunoblotting. **C**. PDPN protein detection (b) in cells by immunocytochemistry with AlexaFuor-murine IgG anti human PDPN (green), nuclei are labelled with DRAG-5 (red). Control (a) is MSU1.1 NC cells (transduced with empty vector). **D**. PDPN mRNA expression level in hypoxia vs normoxia in MSU1.1 PDPN. **E**. PDPN protein expression level in hypoxia vs normoxia in MSU1.1 PDPN. **F**. Quantification of the MDA-MB-231 cells adhesion to MSU1.1 and MSU1.1 PDPN cells surface. MDA-MB-231 cells were counted on the surface of MSU1.1 NC or MSU1.1 PDPN cells (ten representative fields were counted) after flow adhesion in normoxia. * p<0.05, N=3 (mean from ten representative fields). **G**. Effect of CCL21/CCR7 interaction on MDA-MB-231 cell adhesion onto MSU 1.1 PDPN cells in normoxia. **H**. Effect of CCL21/CCR7 interaction on MDA-MB-231 cell adhesion on MSU1.1 PDPN cells in hypoxia. **I**. Effect of CCL21/CCR7 interaction on MDA-MB-231 cell adhesion on MSU1.1 NC cells in normoxia. **J**. Reduction of adhesion of NKL3 cells to MSU1.1 PDPN vs MSU1.1 NC and MSU 1.1.

The role of the CCL21/CCR7 axis in the breast tumor microenvironment was further analysed for the tumor cell interaction with stromal cells, namely the cancer associated fibroblasts. For this purpose the human breast cancer model MDA-MB-231 was chosen for its aggressive properties and expression of CCR7 as shown in Supplementary Figure 2 as compared to the non-aggressive MCF-7 breast cancer cell line. To represent a CAF cell model, MSU1.1 human fibroblastic cells were transduced to express podoplanin (MSU1.1 PDPN) as described in Materials and Methods. As shown in Figure 5, PDPN was found to be expressed both at the mRNA level (Figure 5A) and the protein level (Figure 5B) and in cells, by immunochemistry (Figure 5C). Podoplanin expression is modulated by hypoxia as shown in (Figure 5D, 5E). The mRNA level of PDPN was higher (1.8 fold) upon hypoxia treatment (Figure 5D), and confirmed at the protein level by immunoblotting (Figure 5E). The role of PDPN in the adhesion process of MDA-MB-231 cells to CAFs was studied. Adhesion was reduced by 50%, when MSU1.1 cells expressed PDPN as compared to the control cells transduced by the empty vector, as shown in Figure 5F, when experiments were conducted under flow conditions. Similar to NKL3 cell adhesion processes, MDA-MB-231 adhered more efficiently in normoxia (Figure 5G) than in hypoxia (Figure 5H) on MSU1.1 PDPN.

Upon pre-incubation of the MSU1.1 PDPN in the presence of CCL21 no significant change was observed in the adhesion when performed in normoxia (Figure 5G) while it significantly increased the adhesion of MDA-MB-231 cells in hypoxia (Figure 5H). Under both oxygen tension conditions, adhesion was inhibited by pre-incubation of the cancer cells MDA-MB-231 in the presence of neutralizing anti-CCR7 antibodies (Figure 5G, 5H) which bound efficiently to MDA-MB-231 (Supplementary Figure 2).

This effect is obtained under hypoxic conditions (Figure 5H). Indeed, when MSU1.1 PDPN cells were pre-incubated with exogenous CCL21, the adhesion level of MDA-MB-231 cells was clearly (2 fold) increased (Figure 5H). This increased adhesion effect which was displayed after CCL21 treatment only in hypoxia, was totally inhibited upon blocking the CCR7 receptors on the MDA-MB-231 cancer cells (Supplementary Figure 2) by pre-incubation with anti-CCR7 neutralizing antibodies (Figure 5H). This shows the involvement of the same CCL21/CCR7 axis in the tumor cell to cancer associated fibroblast recognition.

Figure 5I displays the same type of experiment on non PDPN expressing MSU1.1 fibroblasts, in normoxia, pointing to the higher adhesion activity of MDA-MB-231 cells. In normoxia, the CCR7 inhibition effect indicates that the CCR7/CCL21 axis is involved in the recognition similar to PDPN expressing fibroblasts in normoxia (Figure 5G). Indeed, CCL21 has no effect on MSU1.1 adhering capacity, thus pointing to a PDPN/CCL21 interaction, as evidenced in hypoxia, on CAFs. The data obtained upon adhesion of MDA-MB-231 on MSU1.1 non expressing PDPN in hypoxia (Supplementary Figure 3) were similar to normoxia, confirming that in CAFs, PDPN can interact with CCL21 to bind CCR7+ cells. It must be pointed out that NKL3 cells, similar to MDA-MB-231 cancer cells, adhere less to PDPN expressing fibroblasts. This confirms an antiadhesive effect of PDPN in the tumor stroma (Figure 5J). While this effect is compensated for by the addition of CCL21 *in vitro*, no increase of the CCL21 level was observed with hypoxia in fibroblasts (Supplementary Figure 4).

### miRNAs expression in normoxia and hypoxia in MSU1.1 and MSU1.1 PDPN

**Figure 6:**
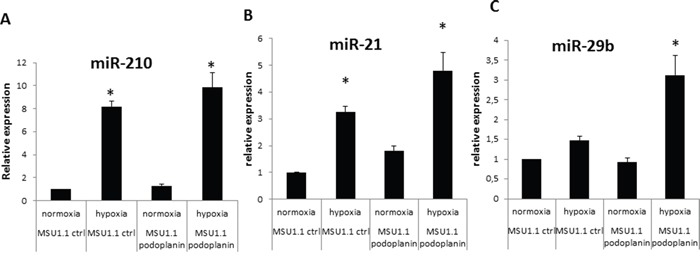
MiRNA expression modulation in MSU 1.1 fibroblasts upon PDPN expression in normoxia and hypoxia **A**. miR-210 increased expression by hypoxia in MSU1.1 and MSU 1.1 PDPN; * p<0.05, N=3. **B**. miR-21 expression increase in MSU1.1 in hypoxia and in MSU1.1 PDPN by hypoxia. * p<0.05, N=3. **C**. hypoxia induced modulation of miR-29b expression in MSU1.1 and in MSU 1.1 PDPN. * p<0.05, N=3.

MSU1.1 and MSU1.1 PDPN cells were incubated in hypoxia and normoxia for 24 h and miR-210, miR-21 and miR-29b expression levels were evaluated using real-time qPCR. In MSU1.1 cells, the level of miR-210 was 8.19 fold higher in hypoxia than in normoxia. In MSU1.1 PDPN cells the level of miR-210 was 7.73 fold higher in hypoxia than in normoxia (Figure 6A). The expression level of miR-21 in MSU1.1 cells was increased 1.66 fold with hypoxia treatment when compared to normoxia. In MSU1.1 PDPN cells this increase was 4.7 fold (Figure 6B). The expression of MiR-29b, a known regulator of PDPN expression, increased 3.06 fold only in MSU1.1 PDPN expressing cells. In MSU1.1 cells, no difference was observed for miR-29b expression in hypoxia vs normoxia.

## DISCUSSION

One of the main pitfalls of anticancer immunotherapeutic strategies is the lack of accessibility of tumor cells to drugs and the hypoxic conditions of the tumor microenvironment which favor cancer stem-like cell selection and immunosuppression [32, 37, 28]. Thus the factors that determine NK cells migration into the tumor microenvironment must be known in order to better understand the mechanism of NK cells infiltration and improve their efficacy in anti-tumor therapies. One approach is to modify the tumor microenvironment in order to allow an efficient NK cell attraction and prevent their inactivation within the tumor by the action of immune checkpoint molecules [38].

In this work two important factors associated with tumor microenvironment were considered since they affect NK cell recruitment, recognition and activity. These factors are hypoxia, which accompanies and helps immunosuppression in solid tumors [32, 39], and chemokines which stimulate endothelial cells in a selective way to induce leukocyte recognition as illustrated in the case of CCL21 [6]. This effect is significant enough for chemokines to be administered into the tumor in various therapeutic approaches [40].

As demonstrated previously, NK cell adhesion and cytotoxicity towards endothelial cells need IL-2 stimulation of lymphocytes (NKL2, NKL3) [34, 36]. Adhesion is specific for lymphoid organ-derived ECs and integrin but it is not selectin dependent [36]. CCL21 is a potent chemoattractant for CCR7+ NK cells and takes part in lymphocyte homing by attracting circulating cells through the establishment of a concentration gradient [6, 36]. In addition to recognition, our research shows that adhesion occurring through CCL21 and its receptor requires dynamic clusterisation of CCR7 in NK cell membranes for tight interaction. But, the basal adhesion level of NK cells to HPLNEC.B3 cells indicates another mechanism in addition to the CCR7-dependent one. As far as the latter is concerned, the involvement of CCL21/CCR7 axis was confirmed by the effect of external addition of CCL21 chemokine and efficient inhibition due to neutralizing anti CCR7- antibodies. The mechanism of CCL21 binding to endothelial cell surfaces is shown here as well as sugar structure specificity. Chondroitin sulfate was determined to be of the glycosaminoglycan type with which CCL21 interacts preferentially. The use of defined oligosaccharides allowed us to demonstrate the specificity for chondroitin sulfate type E. This molecular mechanism was observed in ECs vs NK cell recognition and, noticeably, when compared to normoxia, hypoxia reduced NK cells adhesion, regardless of which treatment was used (CCL21, GAGs or CCR7-neutralizing antibodies). Recognition between ECs and NK cells was considerably lowered by hypoxia although it appeared to be still dependent on the CCL21/ChsE/CCR7 axis. This is in accordance with the observed impairment of NK cell action in tumor hypoxic sites. As other cancer cells, mammary carcinoma cells are able to metastasize into lymph nodes in a process involving the CCL21/CCR7 axis [13]. This implies mobilization from the tumor primary site in which they interact with CAFs which are overexpressing podoplanin. Since CCL21 was found to interact with PDPN [10] the regulation of both molecules may control the entry of CCR7+NK cells and their cytotoxicity in the tumor. Furthermore, it may modulate the movement and escape of CCR7+tumor cells achieving metastasis. Moreover, CCR7 was shown to promote mammary cell tumorigenesis through amplification of stem-like cells [21] which points to the key role played by the CCL21/CCR7 axis in the various steps of mammary tumor evolution.

Overexpression of podoplanin results in a lower interaction of CAFs with carcinoma cells. As shown here, higher PDPN expression is due to hypoxia under which condition, the addition of CCL21 is required to help the CAF/cancer cell interaction. In comparison to normoxia we showed that CCL21 production is not increased upon hypoxia either in CAFs or in carcinoma cells. This confirms the anti-adhesive effect of podoplanin in the tumor microenvironment and its role in favoring CCR7+ carcinoma cell movement and mediating escape. CCR7+ carcinoma cells, as they are less retained in the primary tumor site, might thus be attracted to CCL21+ sites such as peripheral lymph nodes, where the chemokine is presented on endothelial cells as shown in this work. This participates to the understanding of the CCL21/CCR7 impact on the activity and aggressiveness of cancer stem/initiating cells in the hypoxic microenvironment [21, 41, 42, 43]. Moreover, the importance of PDPN in the tumor microenvironment was confirmed by assessing the effect of its expression on micro RNAs (miR-21, miR-210, miR-29b) that are involved in tumor development. MiR-21 is a known oncomiR which regulates tumor suppressors such as PTEN and p53 [30, 31] while miR-210 is the most active hypoxa- and angio- miR. MiR-29b regulates several proangiogenic molecules and podoplanin [40] which, moreover, appears to be positively regulated by hypoxia and co-expressed with miR-21 and miR-210. Since miR-29b is a down regulator of podoplanin, its overexpression in hypoxia in cells, as well as conditions which induce podoplanin expression, suggests a sequential, time-dependent and hypoxia-dependent regulatory effect. This work points to the important part played by hypoxia in the regulation of podoplanin in tumors and its interaction with CCL21. It shows the key role of hypoxia in the restriction of immune cell recruitment favoring immunosuppression. It also demonstrates the part played by CCR7+ tumor cell adhesion to tumor fibroblasts in helping the dissemination of CCR7+, tumor cells which are potentially aggressive cancer stem cells [21, 43].

## MATERIALS AND METHODS

### Cell cultures

Immortalized HPLNEC.B3 endothelial cell lines used in this study display the general characteristics of the *in vivo* endothelium phenotype [e.g. the presence of angiotensin converting enzyme (ACE), the von Willebrand factor and vascular endothelial (VE)-cadherin]. Human microvascular ECs were isolated and immortalized according to the method previously described and patented [35]. The HPLNEC.B3 cells were cultured in OptiMEM with Glutamax-I (Invitrogen, Cergy Pontoise, France) supplemented with 2% MycoPlex FBS (PAA, Les Mureaux, France), 40 μg/ml gentamycin (Invitrogen) and 0.05 μg/ml fungizone (Invitrogen).

The NKL cell line was established from the peripheral blood of a patient with large granular lymphocyte leukemia as previously described [33]. NKL cells were maintained in culture in OptiMEM 1 with Glutamax-I (Invitrogen), supplemented with 3% human AB serum (Institut Jacques Boy), 1% penicillin/streptomycin (Invitrogen), and 0.05 μg/ml fungizone. Additionally, for NKL2 and NKL3 clones [33] culture, 200 U/ml of human interleukin-2 (IL-2) (Roche Diagnostics, Germany) were added.

All the cells were maintained at 37°C in a 5% CO2/95% air atmosphere.

### Hypoxia treatment

Cells were maintained under hypoxic conditions for 24 hours by flowing a 5% CO2, 95% N2 gas mixture in an automated PROOX chamber (C-174, BioSpherix, USA). An 1% oxygen level was controlled by a PROOX sensor (model 110, BioSpherix).

### mAbs and reagents

Mouse IgG2A anti human CCR7 fluorescein-coupled antibodies, mouse IgG2a fluorescein-coupled isotypic control, mouse IgG2A anti human CCR7 neutralizing antibodies, recombinant human CCL21 and human CCL21 ELISA kits were purchased from R&D Systems (UK). Chondroitin sulfate E was obtained from Seikagaku Corp. (Japan). The PKH26GL Red Fluorescent Cell Linker kit and fibronectin were purchased from Sigma-Aldrich (France).

### Expression of CCR7 on NK cells

During all the labelling procedures, cells were maintained at 4°C. 5.10^5^ cells were washed twice with complete phosphate-buffered saline (cPBS) (1 mM CaCl2 and 0.5 mM MgCl2), 0.5% bovine serum albumin (BSA) [weight/volume (w/v)] (Sigma-Aldrich). Then, cells were incubated in the presence of 2.5 μg/ml mouse IgG2A anti human CCR7 fluorescein-coupled antibodies for 1 hour at 4°C. Cells were washed twice with cPBS and analyzed by flow cytometry on a FACS LSR II apparatus (Becton Dickinson, Sunnyvale, CA) using CELLQUEST software (Becton Dickinson).

### Capping of CCR7

NK cells were labelled at 4°C as previously described. Then, receptor rearrangement was visualized by fluorescent microscopy raising the cell temperature from 4°C to room temperature. Cell fluorescence was observed with the use of an Axiovert 200 epifluorescence inverted microscope (Zeiss, Le Pecq, France). Pictures were taken every 5 minutes at room temperature.

### Flow adhesion experiments

Flow adhesion experiments used either a flow chamber (Immunetics, Boston, MA) as previously described [44] or the BioFlux system (Labtech, France). Forty-eight hours before the experiment, HPLNEC.B3 cells were seeded either on polystyrene tissue-culture slides (Nagle Nunc International) or on a 48-well BioFlux plate, according to the manufacturer's instructions. Briefly, channels on the Bioflux plate were coated with 50 μg/ml of fibronectin (Sigma, France). Then, cells were seeded into the channels and 5 hours later, the medium was removed from the outlet well to allow a passive flow of medium. Forty-eight hours after seeding, HPLNEC.B3 cells were either treated or not treated with CCL21 chemokine and/or GAGs. Cells were incubated in basal OptiMEM for 1 hour at 37°C with 17 nM or 50 nM of human recombinant CCL21 on a culture slide and a 48-well Bioflux plate, respectively or with 0.6 μg/ml of chondroitin sulfate E (ChSE, Seikagaku, Japan). When CCL21 was combined with ChSE for treatments, chemokine was pre-incubated 15 minutes at room temperature with ChSE and then added to the cells for 1 hour at 37°C.

For image acquisition, the flow chamber or the Bioflux plate was mounted on an Axiovert 200 epifluorescence inverted microscope (Zeiss) for direct real-time visualization of the dynamic cell adhesion process using a X10 objective. The microscope was coupled to an Axiocam high-resolution numeric camera (Carl Zeiss) attached directly to a computer equipped with the acquisition software Axiovision (Zeiss).

### CCL21 secretion by HPLNEC.B3 cells under normoxia and hypoxia

HPLNEC.B3 cells were cultured for 24 hours under hypoxia or normoxia. Then cells were lysed with lysis buffer (1% Triton X-100; Sigma, Protease Inhibitor Cocktail; Roche). The protein content in each sample was quantified with a BCA-based method (Thermo Fisher Scientific Inc., France).

### GAG biotinylation procedure

GAGs (chondroitin sulfate A, B, C, D, E, heparan sulfate, keratan sulfate and hyaluronic acid, from Seikagaku Corp. (Japan), re-suspended in PBS at 10 mg/ml, were allowed to react under agitation for 24 h at room temperature with 10 mM biotin/LC-hydrazine (Perbio Science, Brebières, France). To remove unreacted biotin-LC-hydrazide, each reaction mixture was applied to a Trisacryl GF05 M (BioSepra, Villeneuve La Garenne, France) column. The fractions containing biotinylated GAGs were detected using a calorimetric method using resorcinol and sulfuric acid [45].

### Kinetic analysis using BIAcore

The experiment was performed on a BIAcoreTM 2000 system (BIAcore AB, Uppsala, Sweden). All experiments were performed at 25°C. Sensor chips SA (BIAcore AB) were used. Chips were first conditioned with three consecutive 1 minute injections of 1 M NaCl in 50 mM NaOH.

Biotinylated GAGs, diluted in HBS (10 mM Hepes, 150 mM NaCl, 3.4 mM EDTA, 0.005% surfactant P20, pH 7.4, BIAcore) containing 0.3 M NaCl, were injected on one flow cell of the chip SA. The first flow cell was used as a negative control. Approximately 100 resonance units (RU) of material were immobilized for each GAG.

The running buffer used for the washing and dissociation phases was PBS-BSA 0.2%.

For the binding assays, different concentrations of chemokines were injected at 30 μl/min for CCL5 and 50 μl/min for CCL21, to eliminate mass transport effects. The sensor chip surface was regenerated with a 1-minute injection of 1 M NaCl. No significant change in the baseline was observed after surface regeneration. Affinity kinetic parameters were determined with BIAevaluation 3.0 software (BIAcore) using a single-site binding model.

## SUPPLEMENTARY MATERIALS FIGURES


