# Supplementary material for: Tumor hypoxia modulates podoplanin/CCL21 interactions in CCR7+ NK cell recruitment and CCR7+ tumor cell mobilization
Source: Oncotarget. 2017 Mar 17;8(19):31876–87. doi: 10.18632/oncotarget.16311 (PMC5458255; doi:10.18632/oncotarget.16311)
Supplement: Supplementary file 1 [file oncotarget-08-31876-s001.pdf]

Tumor hypoxia modulates podoplanin/CCL21 interactions in CCR7+ NK cell recruitment and CCR7+ tumor cell mobilization

SUPPLEMENTARY FIGURES

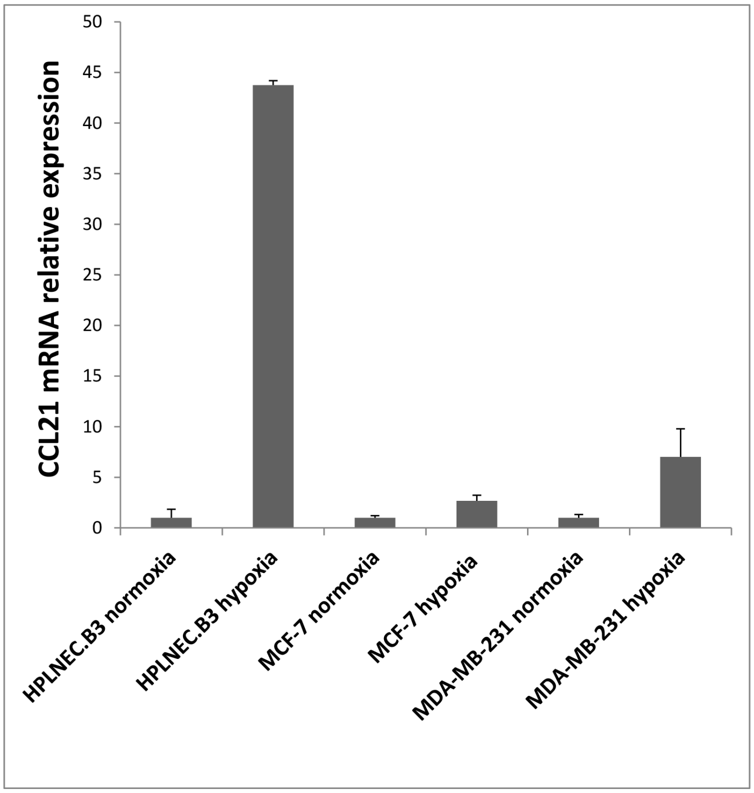

Supplementary Figure 1: CCL21 expression in cell lines upon modulation by hypoxia. Cells were cultured for 24 hours in normoxia or hypoxia (O_2 1%) then RNA were extracted, and quantified by RQPCR.

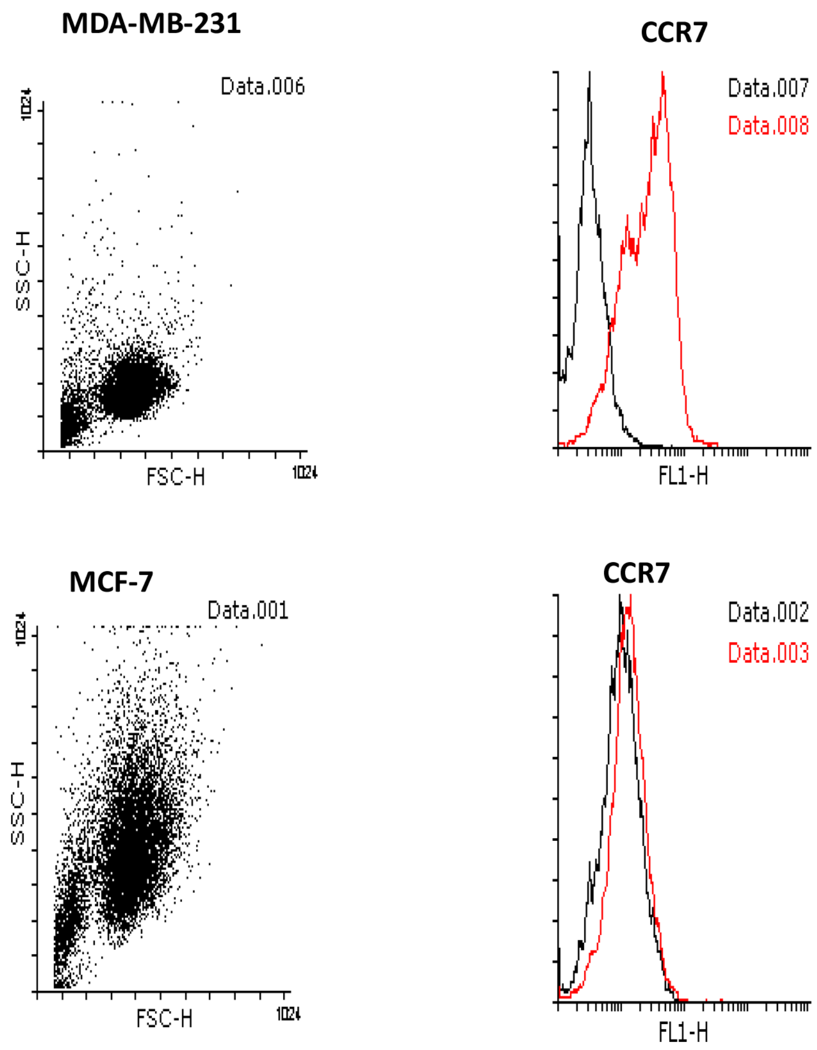

Supplementary Figure 2: Expression of CCR7 on cancer cells MDA-MB-231 and MCF7 cells were labelled by FTC anti CCR7 antibodies at 4°C, 1 h. Labeling was assessed by Flow Cytometry on FACS LSR.

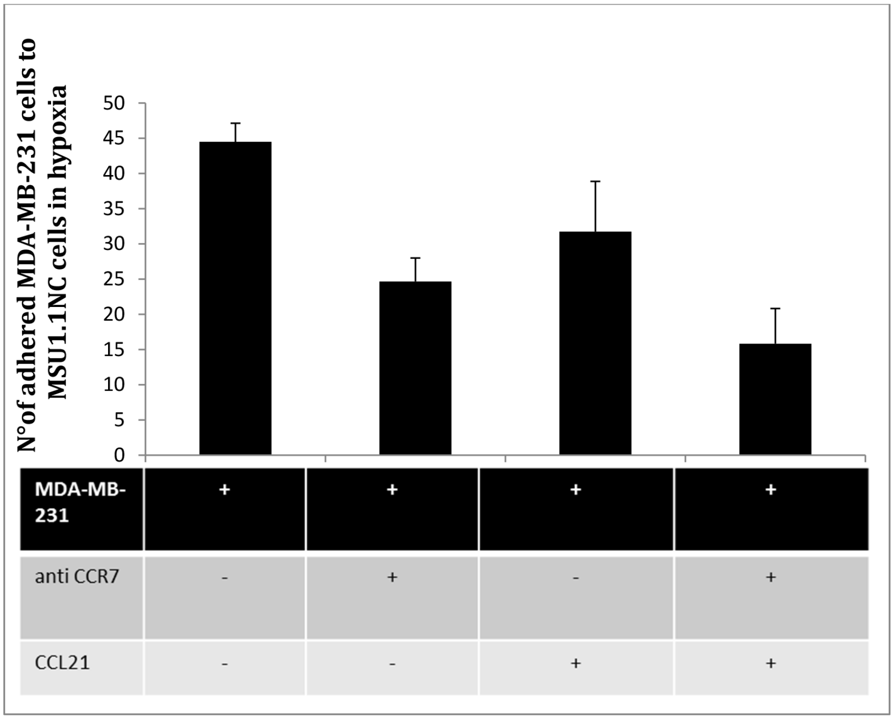

Supplementary Figure 3: CCL21/CCR7 interaction in MDA-MB-231 MSU1.1 NC cells in hypoxia. Quantification of the MDA-MB-231 cells adhesion to MSU1.1 cells surface. MDA-MB-231 cells were counted on the surface of MSU1.1 NC or MSU1.1 PDPN cells (ten representative fields were counted) after flow adhesion in normoxia. * p<0.05, N=3 (mean from ten representative fields).

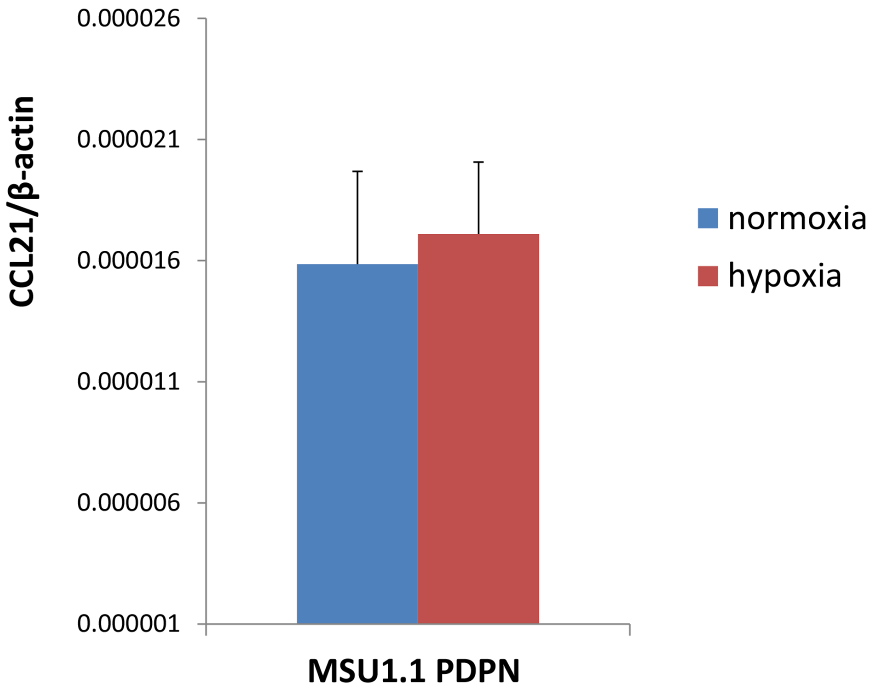

Supplementary Figure 4: Expression of CCL21 in MSU1.1 PDPN fibroblasts upon treatment by hypoxia. Cells were cultured for 24 hours in normoxia or hypoxia (O_2 1%) then RNAs were extracted and quantified by RQPCR.
